# Using CRISPR Interference as a Therapeutic Approach to Treat TGFβ2-Induced Ocular Hypertension and Glaucoma

**DOI:** 10.1167/iovs.62.12.7

**Published:** 2021-09-09

**Authors:** Naga Pradeep Rayana, Chenna Kesavulu Sugali, Jiannong Dai, Michael Peng, Shaohui Liu, Yucheng Zhang, Jun Wan, Weiming Mao

**Affiliations:** 1 Eugene & Marilyn Glick Eye Institute, Department of Ophthalmology, Indiana University School of Medicine, Indianapolis, Indiana, United States; 2 Department of Medical and Molecular Genetics, Indiana University School of Medicine, Indianapolis, Indiana, United States; 3 Center for Computational Biology and Bioinformatics, Indiana University School of Medicine, Indianapolis, Indiana, United States; 4 Department of BioHealth Informatics, Indiana University School of Informatics and Computing, Indiana University—Purdue University Indianapolis, Indianapolis, Indiana, United States; 5 Department of Biochemistry and Molecular Biology, Indiana University School of Medicine, Indianapolis, Indiana, United States; 6 Department of Pharmacology and Toxicology, Indiana University School of Medicine, Indianapolis, Indiana, United States

**Keywords:** TGFβ2, CRISPR interference, ocular hypertension, glaucoma, mouse model

## Abstract

**Purpose:**

Primary open angle glaucoma (POAG) is a leading cause of blindness worldwide with elevated intraocular pressure (IOP) as the most important risk factor. POAG IOP elevation is due to pathological changes in the trabecular meshwork (TM). Elevated TGFβ2 contributes to these changes and increases IOP. We have shown that histone hyperacetylation is associated with TGFβ2 elevation in the TM. In this study, we determined if clustered regularly interspaced short palindromic repeats (CRISPR) interference could specifically deacetylate histones and decrease TGFβ2 in the TM.

**Methods:**

We tested the efficiency of different promoters in driving KRAB-dCAS9 expression in human TM cells. We also screened and determined the optimal sgRNA sequence in the inhibition of TGFβ2. Chromatin immunoprecipitation-qPCR was used to determine the binding of KRAB-dCAS9. An adenovirus-mediated TGFβ2-induced ocular hypertension (OHT) mouse model was used to determine the effect of the CRISPR interference system in vivo.

**Results:**

We found that the CRISPR interference system inhibited TGFβ2 expression in human TM cells, and properly designed sgRNA targeted the promoter of the TGFβ2 gene. Using sgRNA targeting the CMV promoter of the Ad5-CMV-TGFβ2 viral vector, we found that lentivirus-mediated KRAB-dCAS9 and sgRNA expression was able to inhibit Ad5-CMV-TGFβ2-induced OHT in C57BL/6J female and male mice eyes. This inhibition of OHT was associated with decreased levels of TGFβ2 and extracellular matrix proteins in the mouse eye.

**Conclusions:**

Our results indicate that CRISPR interference is a useful tool for gene inhibition and may be a therapeutic approach to treat TGFβ2-induced OHT.

Glaucoma is a group of eye diseases which lead to visual impairment and blindness.^1^ Glaucoma is characterized by damage to the optic nerve. Among all types of glaucoma, primary open angle glaucoma (POAG) is the most prevalent type.^1^ Elevated intraocular pressure (IOP) is the major risk factor and treatment target of POAG.^2^ In POAG eyes, an increase in aqueous humor outflow resistance at the trabecular meshwork (TM) at the iridocorneal angle leads to elevated IOP. Elevated IOP initiates damages in retinal ganglion cells at the lamina cribrosa.^3^ Pathological changes in the glaucomatous TM include loss of TM cells, increased TM stiffness, excessive cross-linked actin network formation, compromised TM cell functionality, and aberrant accumulation of extracellular matrix (ECM).^4^

Several POAG-associated factors have been identified.^5^ Among these factors, transforming growth factor-β2 (TGFβ2) is the most well studied. TGFβ2 is a secreted protein, and it is elevated in the TM and aqueous humor of POAG patients.^6^^,^^7^ Studies showed that TGFβ2 induces the expression of ECM and crosslinking enzymes,^8^^,^^9^ induces cross-linked actin networks,^10^ and likely increases TM stiffness. TGFβ2 also induces ocular hypertension (OHT) in perfusion cultured human eyes as well as in in vivo mouse eyes.^11^^,^^12^

Although elevated TGFβ2 is closely associated with OHT and POAG, the etiology of elevated expression of TGFβ2 is unclear. To our best knowledge, no known mutations of TGFβ2 or its receptors associated with POAG have been reported. Therefore, gene editing is not suitable for treating most of the POAG patients due to the lack of gene targets. In contrast, epigenetic regulations may cause high levels of TGFβ2. Epigenetic regulatory mechanisms control gene expression without altering the genomic sequence. They modify DNA and its associated proteins (e.g., histones and transcriptional factors) either chemically (e.g., methylation and acetylation) or structurally (unwinding, compacting, looping, alternative splicing, etc.).^13^ These changes facilitate or inhibit gene transcription.

Our published studies suggest that elevated TGFβ2 in the glaucomatous TM may result from histone hyperacetylation at the TGFβ2 promoter region.^14^ We found that glaucomatous TM cells have a higher level of histone acetylation compared to nonglaucomatous TM cells.^14^ Treatment with histone deacetylase (HDAC) inhibitors increased histone acetylation in the TGFβ2 promoter region as well as cellular and secreted TGFβ2 in human primary TM cells.^14^ In addition, perfusion cultured bovine eyes receiving HDAC inhibitors developed OHT.^14^ Therefore, modification of histone acetylation is a potential therapeutic approach for lowering TGFβ2 in the glaucomatous TM.

There are many HDAC inhibitors available. However, these agents are not gene-specific, and they modify several types of histones at many genomic locations. Promoter-specific HDAC modification was not possible until the introduction of the clustered regularly interspaced short palindromic repeats (CRISPR) interference technology. The CRISPR interference system is based on the RNA guided mutant CAS9 (dCAS9).^15^ Unlike the CAS9 protein, which is able to cleave DNA, the dCAS9 protein lacks DNA endonuclease activity.^15^ With the help of single guide RNA (sgRNA), dCAS9 binds to a specific locus (usually the promoter region), blocks the binding and/or passaging of RNA polymerase, and silences the gene of interest.^16^^,^^17^ The effect of dCAS9 can be further improved by fusion with the Kruppel-associated box (KRAB) domain forming the KRAB-dCAS9 complex.^15^^,^^18^ KRAB is a member of transcriptional regulators in higher vertebrates, with 400 zinc finger proteins and functions as a histone deacetylase.^19^ Since the KRAB-dCAS9 addresses the potential etiology of elevated TGFβ2 in the glaucomatous TM, we investigated if this system is able to specifically deacetylate the TGFβ2 gene promoter, lower TGFβ2 in the TM, and inhibit TGFβ2-induced OHT.

## Methods

### Vector Construction

The pHR-SFFV-KRAB-dCAS9-P2A-mCherry vector was a gift from Jonathan Weissman (Addgene plasmid #60954; http://n2t.net/addgene:60954; RRID: Addgene_60954; Watertown, MA, USA).^17^ This vector was used to express KRAB-dCAS9 in most of the assays. The other two vectors, pLVX-EF1α-KRAB-dCAS9-P2A-mCherry and pLVX-CMV-KRAB-dCAS9-P2A-mCherry, were also used in some of the assays. The pLVX-EF1α-KRAB-dCAS9-P2A-mCherry vector was constructed by inserting the KRAB-dCAS9-P2A-mCherry sequence with a stop codon at the 3’ end into the pLV-EF1α-mCherry-N1 vector (Takara Bio USA, Ann Arbor, MI, USA) so that the mCherry gene in the backbone was not expressed. The pLVX-CMV-KRAB-dCAS9-P2A-mCherry vector was constructed by inserting the KRAB-dCAS9-P2A-mCherry sequence with a stop codon into the pLVX-AcGFP-N1 vector (Takara Bio USA) so that the AcGFP gene in the backbone was not expressed. Both sequences were inserted at the EcoRI (5’) and BamHI (3’) sites using the in-Fusion HD cloning kit (Takara Bio USA). The pLVX-CMV-ΔhTGFβ2^C226S/C228S^-AcGFP vector was constructed by subcloning the ΔhTGFβ2^C226S/C228S^ coding sequence from the pacAd5-CMV-ΔhTGFβ2^C226S/C228S^ vector (A kind gift from Alcon laboratory, Fort Worth, TX, USA) into the EcoRI (5’) and BamHI (3”) sites of the pLVX-AcGFP-N1 vector so that the ΔhTGFβ2^C226S/C228S^-AcGFP fusion protein will be expressed. The same in-Fusion HD cloning kit was used for subcloning.

The sgRNAs targeting the human TGFβ2 promoter region were designed using two different online tools. Four sgRNAs were designed and selected using https://zlab.bio/guide-design-resources, and the other 20 sgRNAs were designed and selected using http://crispor.tefor.net/.^20^ The off-target score was used as the criteria for selection.^21^ These sgRNAs were cloned into the Lentiguide-puro vector (A gift from Feng Zhang; Addgene plasmid # 52963; http://n2t.net/addgene:52963; RRID:Addgene_52963) with a U6 promoter.^22^ A non-targeting sgRNA (NT sgRNA) expression vector was used as a negative control. It (BRDN0001149198) was a gift from John Doench & David Root (Addgene plasmid # 80248; http://n2t.net/addgene:80248; RRID:Addgene_80248).^23^ A similar approach was used to design the sgRNAs targeting the CMV promoter region of the Ad5-CMV-ΔhTGFβ2^C226S/C228S^ adenovirus (Vector Biolabs, Malvern, PA, USA). All oligos for sgRNA subcloning were synthesized by Sigma-Aldrich (St. Louis, MO, USA).

Oligos for TGFβ2 targeting sgRNAs:

**Table:** 

TGFβ2 sgRNA 1 sense	5’-CACCGAGCTCTCCCCGAACCGTTGA-3’
TGFβ2 sgRNA 1 antisense	5’-AAACTCAACGGTTCGGGGAGAGCTC-3’
TGFβ2 sgRNA 15 sense	5’-CACCGGATAACATCACGATCTTGCC-3’
TGFβ2 sgRNA 15 antisense	5’-AAACGGCAAGATCGTGATGTTATCC-3’
TGFβ2 sgRNA 5 sense	5’-CACCGCTCGTGGTCTAAGTAACGAG-3’
TGFβ2 sgRNA 5 antisense	5’-AAACCTCGTTACTTAGACCACGAGC-3’
TGFβ2 sgRNA 23 sense	5’-CACCGACTTATAAATCTCCCCTCCC-3’
TGFβ2 sgRNA 23 antisense	5’-AAACGGGAGGGGAGATTTATAAGTC-3’
TGFβ2 sgRNA 30REV sense	5’-CACCGAGCTCTCCCCGAACCGTTGA-3’
TGFβ2 sgRNA 30REV antisense	5’-AAACTCAACGGTTCGGGGAGAGCTC-3’
TGFβ2 sgRNA 148REV sense	5’-CACCGACATCACGATCTTGCCGGGG-3’
TGFβ2 sgRNA 148REV antisense	5’-AAACCCCCGGCAAGATCGTGATGTC-3’
TGFβ2 sgRNA 71FORW sense	5’-CACCGCTCGTGGTCTAAGTAACGAG-3’
TGFβ2 sgRNA 71 FORW antisense	5’- AAACCTCGTTACTTAGACCACGAGC-3’
TGFβ2 sgRNA 43 FORW sense	5’-CACCGCCACACTCCCTCAACGGTTC-3’
TGFβ2 sgRNA 43 FORW antisense	5’-AAACGAACCGTTGAGGGAGTGTGGC-3’
TGFβ2 sgRNA 44 FORW sense	5’-CACCGCACACTCCCTCAACGGTTCG-3’
TGFβ2 sgRNA 44 FORW antisense	5’-AAACCGAACCGTTGAGGGAGTGTGC-3’
TGFβ2 sgRNA 147 REV sense	5’-CACCGCATCACGATCTTGCCGGGGA-3’
TGFβ2 sgRNA 147 REV antisense	5’-AAACTCCCCGGCAAGATCGTGATGC-3’
TGFβ2 sgRNA 146 REV sense	5’-CACCGATCACGATCTTGCCGGGGAG-3’
TGFβ2 sgRNA 146 REV antisense	5’-AAACCTCCCCGGCAAGATCGTGATC-3’
TGFβ2 sgRNA 42 FORW sense	5’-CACCGTCCACACTCCCTCAACGGTT-3’
TGFβ2 sgRNA 42 FORW antisense	5’-AAACAACCGTTGAGGGAGTGTGGAC-3’
TGFβ2 sgRNA 151 REV sense	5’-CACCGATAACATCACGATCTTGCCG-3’
TGFβ2 sgRNA 151 REV antisense	5’-AAACCGGCAAGATCGTGATGTTATC-3’
TGFβ2 sgRNA 202 FORW sense	5’-CACCGAGCAGAAGGTTCGCTCCGAG-3’
TGFβ2 sgRNA 202 FORW antisense	5’-AAACCTCGGAGCGAACCTTCTGCTC-3’
TGFβ2 sgRNA 153 REV sense	5’-CACCGAGATAACATCACGATCTTGC-3’
TGFβ2 sgRNA 153 REV antisense	5’-AAACGCAAGATCGTGATGTTATCTC-3’
TGFβ2 sgRNA 31 REV sense	5’-CACCGAGCTCTCCCCGAACCGTTG-3’
TGFβ2 sgRNA 31 REV antisense	5’-AAACCAACGGTTCGGGGAGAGCTC-3’

**Table:** 

TGFβ2 sgRNA 89 REV sense	5’-CACCGCTTCAACAAAGTGACGTGCT-3’
TGFβ2 sgRNA 89 REV antisense	5’- AAACAGCACGTCACTTTGTTGAAGC-3’
TGFβ2 sgRNA 152 REV sense	5’-CACCGATAACATCACGATCTTGCC-3’
TGFβ2 sgRNA 152 REV antisense	5’-AAACGGCAAGATCGTGATGTTATC-3’
TGFβ2 sgRNA 23 REV sense	5’-CACCGCCCGAACCGTTGAGGGAGTG-3’
TGFβ2 sgRNA 23 REV antisense	5’-AAACCACTCCCTCAACGGTTCGGGC-3’
TGFβ2 sgRNA 4 REV sense	5’-CACCGTGGAAATGAGGACCGCTGT-3’
TGFβ2 sgRNA 4 REV antisense	5’-AAACACAGCGGTCCTCATTTCCAC-3’
TGFβ2 sgRNA 110 FORW sense	5’-CACCGCTAGCACGTCACTTTGTTGA-3’
TGFβ2 sgRNA 110 FORW antisense	5’-AAACTCAACAAAGTGACGTGCTAGC-3’
TGFβ2 sgRNA 197 REV sense	5’-CACCGGAGCTTCTGGAGCTCCGCT-3’
TGFβ2 sgRNA 197 REV antisense	5’-AAACAGCGGAGCTCCAGAAGCTCC-3’
TGFβ2 sgRNA 154 FORW sense	5’-CACCGACTTATAAATCTCCCCTCCC-3’
TGFβ2 sgRNA 154 FORW antisense	5’-AAACGGGAGGGGAGATTTATAAGTC-3’
TGFβ2 sgRNA 209 REV sense	5’-CACCGTTTCTCTTGTCAGGAGCTTC-3’
TGFβ2 sgRNA 209 REV antisense	5’-AAACGAAGCTCCTGACAAGAGAAA-3’

Oligos for CMV promoter targeting sgRNAs:

**Table:** 

CMVIE_guideRNA47 sense	5’-CACCGCTTACGGTAAATGGCCCGCC-3’
CMVIE_guideRNA47 antisense	5’-AAACGGCGGGCCATTTACCGTAAGC-3’
CMVIE_guideRNA100 sense	5’-CACCGAGTCCCTATTGGCGTTACTA-3’
CMVIE_guideRNA100 antisense	5’-AAACTAGTAACGCCAATAGGGACTC-3’
CMVIE_guideRNA195 sense	5’-CACCGACGTCAATAGGGGGCGTACT-3’
CMVIE_guideRNA195 antisense	5’-AAACAGTACGCCCCCTATTGACGTC-3’
CMVIE_guideRNA468 sense	5’-CACCGCTACCGCCCATTTGCGTCAA-3’
CMVIE_guideRNA468 antisense	5’-AAACTTGACGCAAATGGGCGGTAGC-3’

### Lentiviral Vector Production and Purification

The Lenti-X packaging single shot (VSV-G) kit (Takara Bio USA) was used for lentiviral production following the manufacturer's protocol. Lenti-X 293T cells (2.2 × 10^6^) were seeded in a 100 mm dish. When the cells became 60% confluent, they were treated with the transfection reagent and plasmids within the kit. Medium was changed after overnight incubation of cells. Conditioned medium containing lentiviral particles was harvested at 48- and 72-hours post transfection. Pooled conditioned medium was first centrifuged at 600*g* for 5 minutes to remove cellular debris. The supernatant was then ultracentrifuged at 40,000*g* for 3 to 4 hours at 4°C. The viral pellet was suspended in 20% sucrose PBS and stored at −80°C in 10 µl aliquots. Viral titer was determined using the Lenti-X qRT-PCR Titration Kit (Takara Bio USA).

### Transfection

The transfection mixture was prepared so that for each well in a 12 well plate, there were 6.4 µl Attractene transfection reagent (Qiagen, Germantown, MD, USA), 1 µg dCAS9-KRAB expression vector, 1 µg sgRNA expression vector/NT sgRNA expression vector, and 1 ml Opti-MEM (Thermo Fisher Scientific, Waltham, MA, USA). The transformed GTM3 cells (1 × 10^5^) (A kind gift from Alcon laboratory) were seeded into each well in the 12 well plate followed by the addition of the transfection mixture. The next day, culture medium was changed to Opti-MEM containing 10% fetal bovine serum, 1% glutamine, and 1% penicillin/streptomycin (Thermo Fisher Scientific).

### Transduction

Lentiviral vectors were transduced into GTM3 or primary human TM cells at the multiplicity of infection (MOI) of 50 (50 viral particles versus one TM cell) with the addition of SureEntry transduction reagent (Qiagen) at 1:4000 or 1:8000 in Opti-MEM with serum in the 12 well plate. Medium was changed the next day.

Some GTM3 cells were transduced with pLVX-CMV-ΔhTGFβ2^C226S/C228S^-AcGFP lentiviral vectors and then selected with puromycin to establish a transgenic cell line (GTM3-ΔhTGFβ2^C226S/C228S^-AcGFP).

### Quantitative Polymerase Chain Reaction (qPCR)

RNA was extracted from transfected or transduced TM cells using the RNeasy Mini Kit (Qiagen). RNA was quantified using NanoDrop 2000c (Thermo Fisher Scientific). RNA was reverse transcribed into cDNA using the iScript Reverse Transcription Supermix (Bio-Rad, Hercules, CA. USA), followed by qPCR using SsoAdvanced Universal SYBR Green Supermix (Bio-Rad) in the CFX96 thermocycler (Bio-Rad). Conditions for qPCR were 95°C for 10 seconds, 60°C for 30 seconds for 40 cycles, followed by melting curve analysis. GAPDH was used as an internal control. Relative gene expression was calculated using the ΔΔCt method.

Primers:

GAPDH^24^ – Forward: 5’-GGTGAAGGTCGGAGTCA-AC-3′

Reverse: 5′-CCATGGGTGGAATCATATTG-3′

TGFβ2 – Forward: 5′-AGAGTGCCTGAACAACGGATT-3′

Reverse: 5′-CCATTCGCCTTCTGCTCTT-3′

### Western Immunoblotting and Densitometry

Five days after transfection or transduction, GTM3 or primary HTM cells were used for protein extraction using the M-PER Mammalian Extraction Reagent (Thermo Fisher Scientific). Equal amount of protein was mixed with 6× Laemmli buffer, boiled, and electrophoresed on SDS-PAGE gel. Separated proteins were transferred onto the Immobilon-P PVDF transfer membrane (Millipore, Burlington, MA, USA), blocked with 10% dry milk, and probed with one of the primary antibodies: mouse anti-TGFβ2 (1:500, ab36495; Abcam, Cambridge, UK), mouse anti-dCas9 (1:200, A-9000-100; Epigentek, Farmingdale, NY, USA), or rabbit anti-GAPDH (1:1000, 5174S; Cell Signaling Technology, Danvers, MA, USA) for 2 hours at room temperature. After washing with TBST, the blot was incubated with the secondary horseradish peroxidase (HRP) linked anti-mouse or anti-rabbit antibody (1:2000 or 1:10,000; Cell Signaling Technology) at 4°C overnight. The protein-antibody complex was incubated with the Clarity Max Western ECL substrate (Bio-Rad) or SuperSignal West Femto Maximum Sensitivity Substrate (Thermo Fisher Scientific). Chemiluminescent signal was detected using the ChemiDoc imager (Bio-Rad).

Densitometry was conducted using the “Gels” tool (under the “Analyze” tab) in the ImageJ software (https://imagej.nih.gov/ij/; National Institute of Health, Bethesda, MD, USA). Naive bands were set at “1.00” for comparisons.

### Chromatin Immunoprecipitation (ChIP) Assay

GTM3 cells were seeded in the 100 mm dish. Upon reaching 50% confluency, the cells were transduced with the lentiviral KRAB-dCAS9 expression vector together with the lentiviral TGFβ2 specific sgRNA expression vector or NT sgRNA expression vector (as a negative control) for 4 days. At the end of transduction, the cells were processed using the TruChIP kit (Covaris, Woburn, MA. USA) according to manufacturer's protocol. Briefly, the cells were fixed, and nuclei were purified. Chromatin shearing was performed using the Covaris Sonicator ME220 (Covaris) with optimized conditions of 6-minute run and 1000 cycles per burst with a peak and average power of 75 watts and 11.25 watts. After sonication, the samples were sent to Indiana University Medical Center for Medical Genomics for analysis using the Agilent 2100 bioanalyzer (Agilent Technologies, Santa Clara, CA, USA). We only used samples showing sheared DNA at the range of 200 to500 bp. Further steps including input preparation, DNA-protein complex pull-down, and DNA purification were performed using the Active Motif eChIP kit (Active Motif, Carlsbad, CA, USA) according to manufacturer's protocol. The dCAS9 antibody (#61757, Active Motif), which recognizes both CAS9 and dCAS9, was used to pull down DNA-dCAS9 protein complex. Mouse IgG was also used for pull-down as a negative control. Purified DNA was quantified using qPCR (SsoAdvanced Universal SYBR Green Supermix; Bio-Rad). The fold of enrichment method was used for data analysis.

Primers:

TGFβ2 1 – Forward - 5’-TTTCCACACTCCCTCAA-CGG-3’

Reverse – 5’-TCTCTGAACCACGTGTCTGC-3’

TGFβ2 5 – Forward - 5’-CCCACAGCGGTCCTCAT-TTC-3’

Reverse – 5’-TGCCAGCAGATAACATCACGA-3’

### The Mouse OHT Model

This study followed the ARVO Statement for the Use of Animals in Ophthalmic and Vision Research. All mice studies were approved by the IACUC at Indiana University School of Medicine. Wild type male and female C57BL/6J mice at the age of 5 months were obtained from Jackson Laboratory (Bar Harbor, ME, USA). The mice were housed at the facility at Laboratory Animal Resource Center (Indiana University School of Medicine; Indianapolis, IN) at reverse light cycle (7 AM off and 7 PM on). All mice were acclimated for more than 2 weeks before any studies.

Baseline and postinjection IOPs were measured using the Tonolab Tonometer (Vantaa, Finland) in anesthetized mouse eyes using the SomnoSuite isoflurane mixer (Kent Scientific; Torrington, CT, USA). We used a concentration of isoflurane at 2% to3%, and IOP was measured between 2 PM and 3 PM. Five readings were taken from each eye, recorded, and averaged.

After obtaining baseline IOP, one mouse eye was intracamerally injected with 10^6^ PFU of the pHR-SFFV-KRAB-dCAS9-P2A-mCherry lentiviral vector together with 10^6^ PFU of the Lentiguide-Puro lentiviral vector expressing the CMV promoter specific sgRNA (produced from the CMVIE_guideRNA100 plasmid vector). The fellow eye was intracamerally injected with 10^6^ PFU of the pHR-SFFV-KRAB-dCAS9-P2A-mCherry lentiviral vector together with 10^6^ PFU of the Lentiguide-Puro lentiviral vector expressing NT sgRNA as a control. Injection was performed using a 33 gauge needle and a 10 µl Hamilton syringe (Hamilton Company, Reno, NV, USA). Starting from the next week, IOP was measured once a week throughout the study. Two and a half weeks after initial viral injection, both mouse eyes received an intravitreal injection of Ad5-CMV-ΔhTGFβ2^C226S/C228S^ virus (2.5 × 10^7^ PFU per female mouse eye and 5 × 10^7^ PFU per male mouse eye). All injections were performed under topical anesthesia (0.5% proparacaine eye drops) and general anesthesia (2%–3% isoflurane). Postprocedure treatment included the topical bacitracin ophthalmic antibiotic ointment and analgesics (Metacam; 5 mg/kg; St. Joseph, MO). Mice were left rested on a controlled heating pad until they were fully conscious and returned to the cages. The mice with no induction of OHT in the NT sgRNA treated eyes were excluded.

### H&E Staining and Immunostaining

The mice were euthanized at the end of the study. The enucleated mouse eyes were fixed in 4% paraformaldehyde for 3 hours on ice, washed in PBS, and embedded in paraffin. The embedded eyes were sectioned at 5 µm in thickness. The sections were heated on a slide warmer for 30 minutes, deparaffinized, and hydrated for H&E staining.

Some deparaffinized slides were used for antigen retrieval in Tris-EDTA pH 9.0 buffer in the 2100 antigen retriever (Electron Microscopy Sciences, Hatfield, PA, USA).

The primary mouse antifibronectin antibody (IST-9, for the ED-A fibronectin isoform; 1:100; ab6328, Abcam) or the mouse anti-TGFβ2 antibody (1:100, ab36495; Abcam) were used. Since these were mouse antibodies, we used the M.O.M. immunodetection kit (Vector Laboratories, Burlingame, CA, USA) to minimize nonspecific binding according to manufacturer's protocol. Briefly, after blocking and primary antibody incubation and washing, the sections were incubated with the avidin conjugated secondary antibody (Vector Laboratories) for 10 minutes. After washing, the sections were incubated with Texas Red Avidin D (A-2006-5; Vector Laboratories) or avidin conjugated with Alexa Flour 488 (A21370; Thermo Fisher Scientific). After washing, the sections were mounted using the Prolong Gold mounting medium with DAPI (Thermo Fisher Scientific). Images were taken using the Nikon Eclipse Ti2 inverted microscope (Nikon, Melville, NY, USA) or Zeiss LSM700 Confocal Microscope (Zeiss, White Plains, NY, USA).

## Results

### Selection of sgRNA and the Promoter for KRAB-dCAS9 Expression

We first screened 24 different types of sgRNAs designed to target the region within 100 base pairs of the transcription start site of the TGFβ2 gene. All the sgRNAs including the NT sgRNA were subcloned into the Lentiguide-Puro vector, a 3rd generation lentiviral vector, and were placed downstream of the U6 promoter.^22^^,^^23^

During initial screening, we transfected transformed GTM3 cells with the sgRNA expression vector (plasmid) and the pHR-SFFV-KRAB-dCAS9-P2A-mCherry vector (plasmid).^17^ The NT sgRNA vector (plasmid) was used as a control.^23^ qPCR was used to determine the efficacy of TGFβ2 inhibition. We found that among the 24 sgRNAs, four sgRNAs (TGFβ2 sgRNA 15, TGFβ2 sgRNA 23, TGFβ2 sgRNA 42, and TGFβ2 sgRNA 43) consistently showed inhibition of TGFβ2 when coexpressed with KRAB-dCAS9 (data not shown).

**Figure 1.**
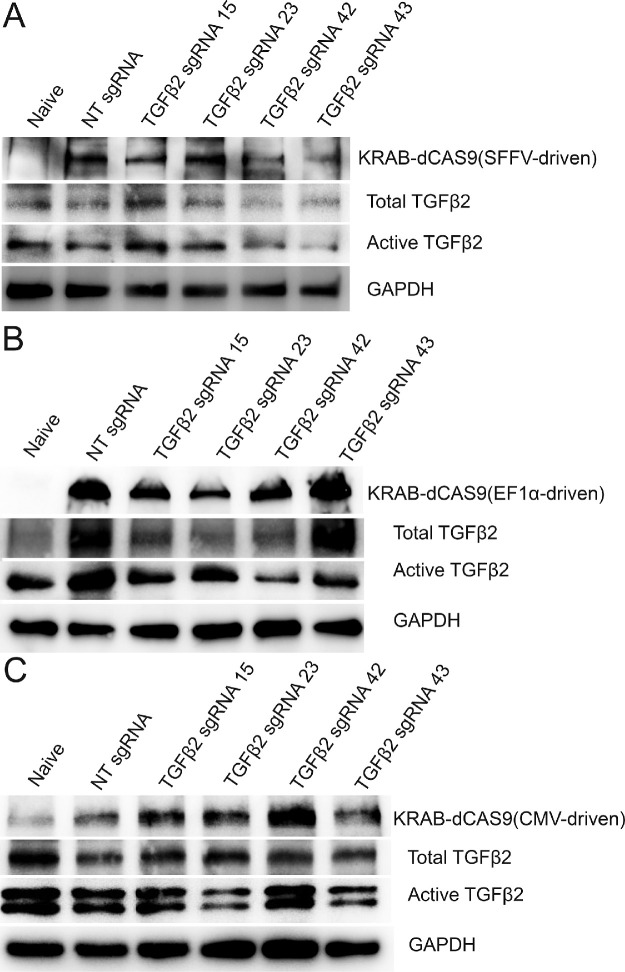
Inhibition of TGFβ2 using different KRAB-dCAS9 expression vectors and sgRNAs in GTM3 cells. GTM3 cells were transfected with pHR-SFFV-KRAB-dCAS9-P2A-mCherry (**A**), pLVX-EF1α-KRAB-dCAS9-P2A-mCherry (**B**), or pLVX-CMV-KRAB-dCAS9-P2A-mCherry (**C**) vectors (plasmids) together with the Lentiguide-Puro vector (plasmid) containing indicated sgRNA expression cassettes. Four days after transfection, whole cell lysates were used for Western immunoblotting. GAPDH was used as a loading control. Naive: TM cells that were not subject to any treatment. NT: nontargeting.

After sgRNA screening, we prepared lentiviruses for the four sgRNAs or NT sgRNA as a control. We also prepared three lentiviruses that express KRAB-dCAS9 using the SFFV, EF1α, or CMV promoter. To determine which promoter is suitable for TM cells, we transduced GTM3 cells with the Lentiguide-Puro lentivirus expressing one of the four TGFβ2 sgRNAs or the NT sgRNA (as a control) together with one of the three KRAB-dCAS9 expressing lentiviruses. Four days after transduction, whole cell lysates were harvested for WB. We found that the SFFV promoter (Fig. 1A) was better than EF1α (Fig. 1B) in the inhibition of both total and active TGFβ2. In contrast, the CMV promoter did not show obvious inhibition (Fig. 1C). Also, we found that TGFβ2 sgRNA 42 showed more consistent TGFβ2 inhibition than the other three sgRNAs (Fig. 1 and Supplementary Table S1).

### KRAB-dCAS9+sgRNA inhibited TGFβ2 Expression in Primary HTM Cells

**Figure 2.**
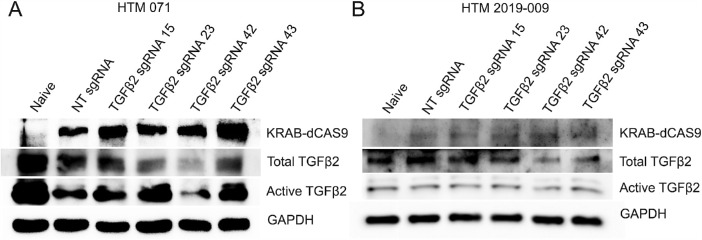
Inhibition of TGFβ2 in primary HTM cells using KRAB-dCAS9 and sgRNA lentiviral expression vectors. Two primary HTM cells strains, HTM 071 (**A**) and HTM 2019-009 (**B**) were transduced with the pHR-SFFV-KRAB-dCAS9-P2A-mCherry lentiviral vector and the Lentiguide-Puro lentiviral vector containing indicated sgRNA expression cassettes. Four days after transduction, whole cell lysates were used for Western immunoblotting. GAPDH was used as a loading control. Naive: TM cells that were not subject to any treatment. NT: nontargeting.

Primary HTM cells (HTM 071: Fig. 2A, and HTM 2019-009: Fig. 2B) were grown to 80% confluency and cotransduced with pHR-SFFV-KRAB-dCAS9-P2A-mCherry (lentivirus) together with Lentiguide-Puro-NT sgRNA/TGFβ2 sgRNA (lentivirus) in the 12 well plate. Four days post transduction, whole cell lysate was collected for Western immunoblotting (WB). The results showed a decrease in the expression of total and active TGFβ2 in cells cotransduced with KRAB-dCAS9 and TGFβ2 sgRNA 42 (Fig. 2 and Supplementary Table S2).

### KRAB-dCAS9 Were Enriched at the TGFβ2 Gene Promoter Region With sgRNA 42

To determine the specificity of TGFβ2 sgRNA 42, we performed ChIP-qPCR assays. Since primary HTM cells grow slowly with limited passage numbers which cannot meet the requirement of ChIP studies (they require a lot of cells), we conducted this assay in transformed GTM3 cells.

**Figure 3.**
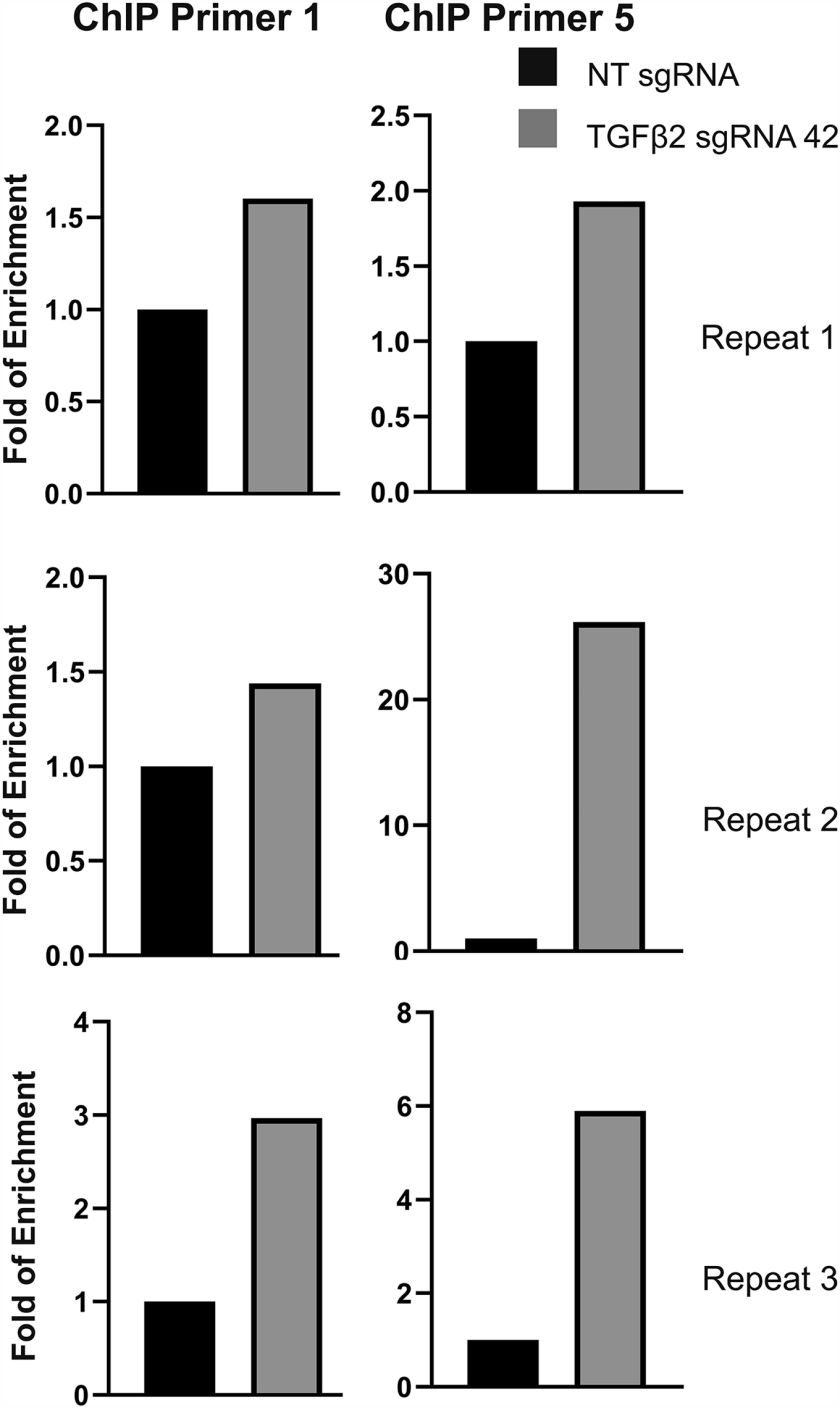
Binding of KRAB-dCAS9 to the promoter region of TGFβ2. GTM3 cells were transduced with pHR-SFFV-KRAB-dCAS9-P2A-mCherry lentiviral vector and the Lentiguide-Puro lentiviral vector containing indicated sgRNA expression cassettes. After ChIP assay using anti-dCAS9 antibody or IgG, purified DNA was used for qPCR using two primer sets (Primer 1 and 5) and fold of enrichment was calculated and shown. Three independent repeats were conducted.

GTM3 cells were cotransduced with the KRAB-dCAS9 ex-pressing lentivirus (pHR-SFFV-KRAB-dCAS9-P2A-mCherry) together with the TGFβ2 sgRNA 42 or NT sgRNA expressing lentivirus (Lentiguide-Puro). Different primer sets were designed targeting the TGFβ2 promoter region, and two primer sets showed reliable amplification using qPCR after immunoprecipitation with the anti-dCas9 antibody or IgG. The experiments were repeated independently three times (all in GTM3 cells), and we consistently observed enrichment of TGFβ2 promoter region genomic DNA (Fig. 3). Our ChIP-qPCR data suggested that there was a KRAB-dCAS9 binding at the TGFβ2 promoter region with the help of TGFβ2 sgRNA 42.

### CRISPR Interference Inhibited Adenovirus-Mediated TGFβ2 Expression In Vitro

We initially planned to induce OHT in mouse eyes using a lentivirus (pLVX-CMV- ΔhTGFβ2^C226S/C228S^) that overexpresses ΔhTGFβ2^C226S/C228S^,^12^ and then inhibit ΔhTGFβ2^C226S/C228S^ expression by using KRAB-dCAS9 and an sgRNA designed against the CMV promoter that drives ΔhTGFβ2^C226S/C228S^ expression. Since lentiviruses integrate the expression cassette into the host genome, we believed that this approach would better mimic the disease status. However, we did not observe IOP elevation after either intravitreal or intracameral injection (data reported in another study). Therefore, we decided to elevate mouse IOP using the well-established Ad5-CMV-ΔhTGFβ2^C226S/C228S^ adenovirus^12^ and use the same strategy to inhibit ΔhTGFβ2^C226S/C228S^ expression (KRAB-dCAS9 and an sgRNA designed against the CMV promoter).

**Figure 4.**
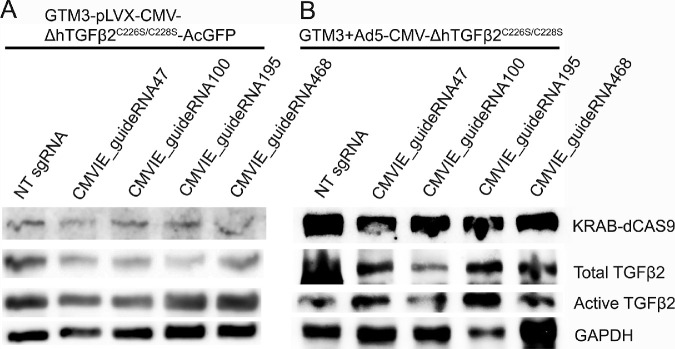
CRISPR interference inhibited TGFβ2 expression in GTM3 cells. A transgenic GTM3 cell line (GTM3-ΔhTGFβ2^C226S/C228S^-AcGFP; this line was established for the initial plan) that expresses ΔhTGFβ2^C226S/C228S^-AcGFP (**A**) and some GTM3 cells that were transduced with the Ad5-CMV-ΔhTGFβ2^C226S/C228S^ viral vector (**B**) were further transduced with the pHR-SFFV-KRAB-dCAS9-P2A-mCherry lentiviral vector and the Lentiguide-Puro lentiviral vector containing indicated sgRNA expression cassettes. Four days post transfection, whole cell lysate was harvested for Western immunoblotting. GAPDH was used as a loading control.

Before in vivo studies, we used GTM3 cells for sgRNA screening. A transgenic cell line (GTM3-ΔhTGFβ2^C226S/C228S^-AcGFP; this line was established for the initial plan) that expresses ΔhTGFβ2^C226S/C228S^ (Fig. 4A) and some GTM3 cells that were transduced with the Ad5-CMV-ΔhTGFβ2^C226S/C228S^ viral vector (Fig. 4B) were further transduced with the KRAB-dCAS9 expressing lentivirus (pHR-SFFV-KRAB-dCAS9-P2A-mCherry) together with the lentivirus (Lentiguide-Puro) expressing NT sgRNA or sgRNA designed for the CMV promoter. Four days after transduction, proteins were extracted for WB. A consistent decrease in the expression of TGFβ2 was observed in cells transduced with CMVIE_guideRNA100 (Fig. 4 and Supplementary Table S3).

### CRISPR Interference Inhibited Ad5-CMV-ΔhTGFβ2^C226S/C228S^ Induced OHT in Mouse Eyes

After sgRNA screening, we determined if KRAB-dCAS9 and CMVIE_guideRNA100 (sgRNA) were able to inhibit Ad5-CMV-ΔhTGFβ2^C226S/C228S^ induced OHT in C57BL/6J mouse eyes. Both male and female mice at the age of 5 months were used. In another study (under review), we found that male mice required a higher dose of Ad5-CMV-ΔhTGFβ2^C226S/C228S^ (5 × 10^7 PFU/eye) to develop OHT compared to female mice (2.5 × 10^7 PFU/eye). Therefore, the indicated doses were used in this study.

**Figure 5.**
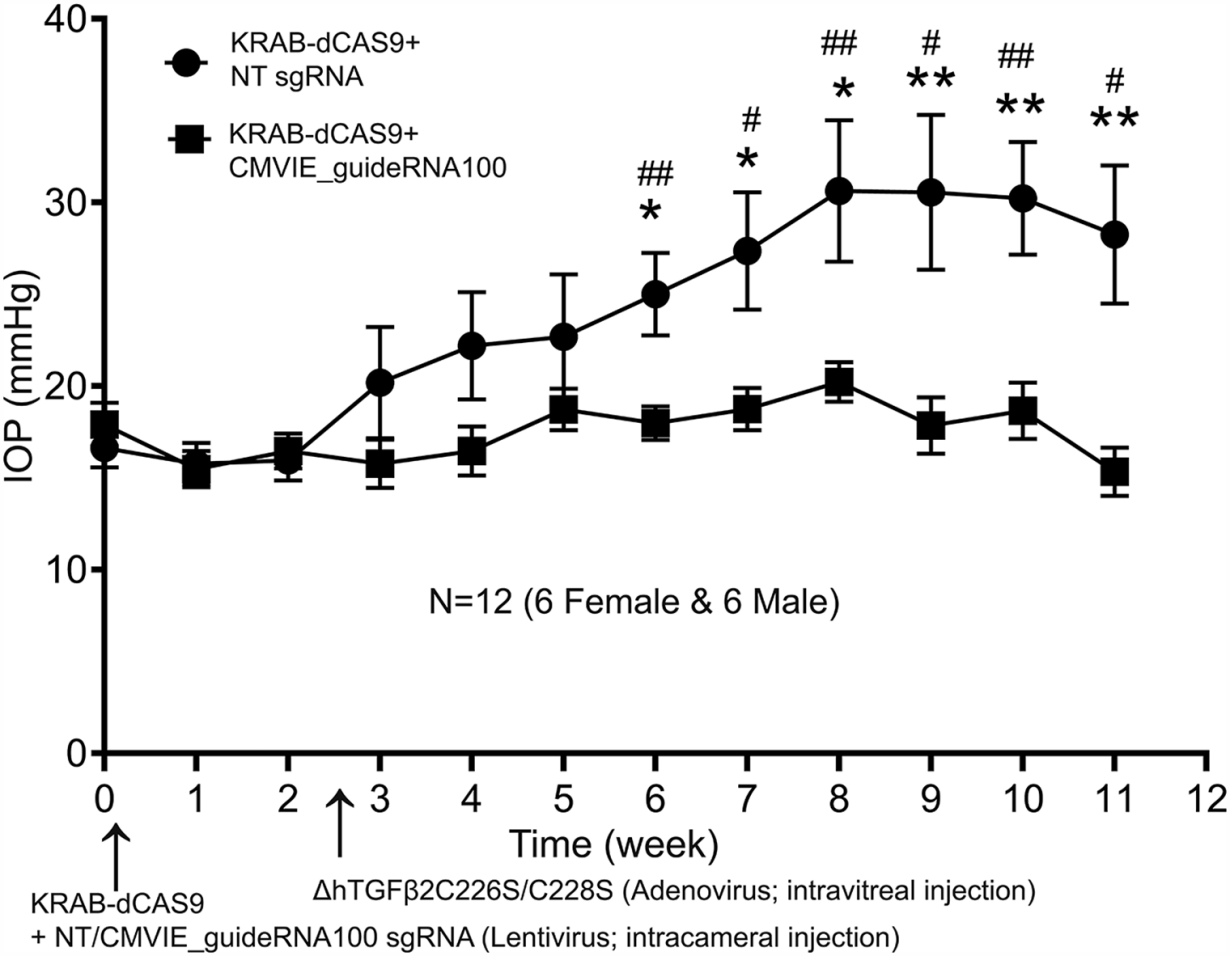
CRISPR interference inhibited TGFβ2-induced OHT in C57BL/6J mouse eyes. Male (*N* = 6) and female (*N* = 6) C57BL/6J mice at the age of 5 months were first intracamerally injected with pHR-SFFV-KRAB-dCAS9-P2A-mCherry and Lentiguide-Puro-CMVIE_guideRNA100 lentiviral vectors in one eye as well as pHR-SFFV-KRAB-dCAS9-P2A-mCherry and Lentiguide-Puro-NT sgRNA in the fellow eye. The mice were then intravitreally injected with the Ad5-CMV-ΔhTGFβ2^C226S/C228S^ adenoviral vector in both the eyes. *: paired *t*-test between eyes. #: One-way ANOVA of CMVIE_guideRNA100 mouse IOP compared to baseline IOP at week 0. * or #: *P* < 0.05, ** or ##: *P* < 0.01. *Dots/squares* and *error bars*: mean and standard error of the mean (SEM).

After acclimation of mice, we intracamerally injected one eye with KRAB-dCAS9 and CMVIE_guideRNA100 expressing lentiviruses. We also injected the fellow eye with KRAB-dCAS9 and NT sgRNA expressing lentiviruses as a control. About 2.5 weeks after lentiviral injection, we intravitreally injected Ad5-CMV-ΔhTGFβ2^C226S/C228S^ viruses into both eyes to induce OHT. A significant increase in IOP was observed in NT sgRNA treated eyes compared to the CMVIE_guideRNA100 treated eyes 3 weeks after adenovirus injection (6 weeks from baseline establishment) (Paired *t*-test; *N* = 12; *P* < 0.05 or 0.01) (Fig. 5). This IOP elevation was also significant compared to its own baseline (one-way ANOVA; *N* = 12; *P* < 0.05 or 0.01) (Fig. 5). In contrast, the CMVIE_guideRNA100 treated eyes did not develop OHT throughout the study (Fig. 5).

Further, we enucleated mouse eyes and fixed them for hematoxylin and eosin (H&E) staining as well as immunostaining to determine the morphology as well as the expression of TGFβ2 and fibronectin isoform EDA (FN-EDA) of/in the TM region.

**Figure 6.**
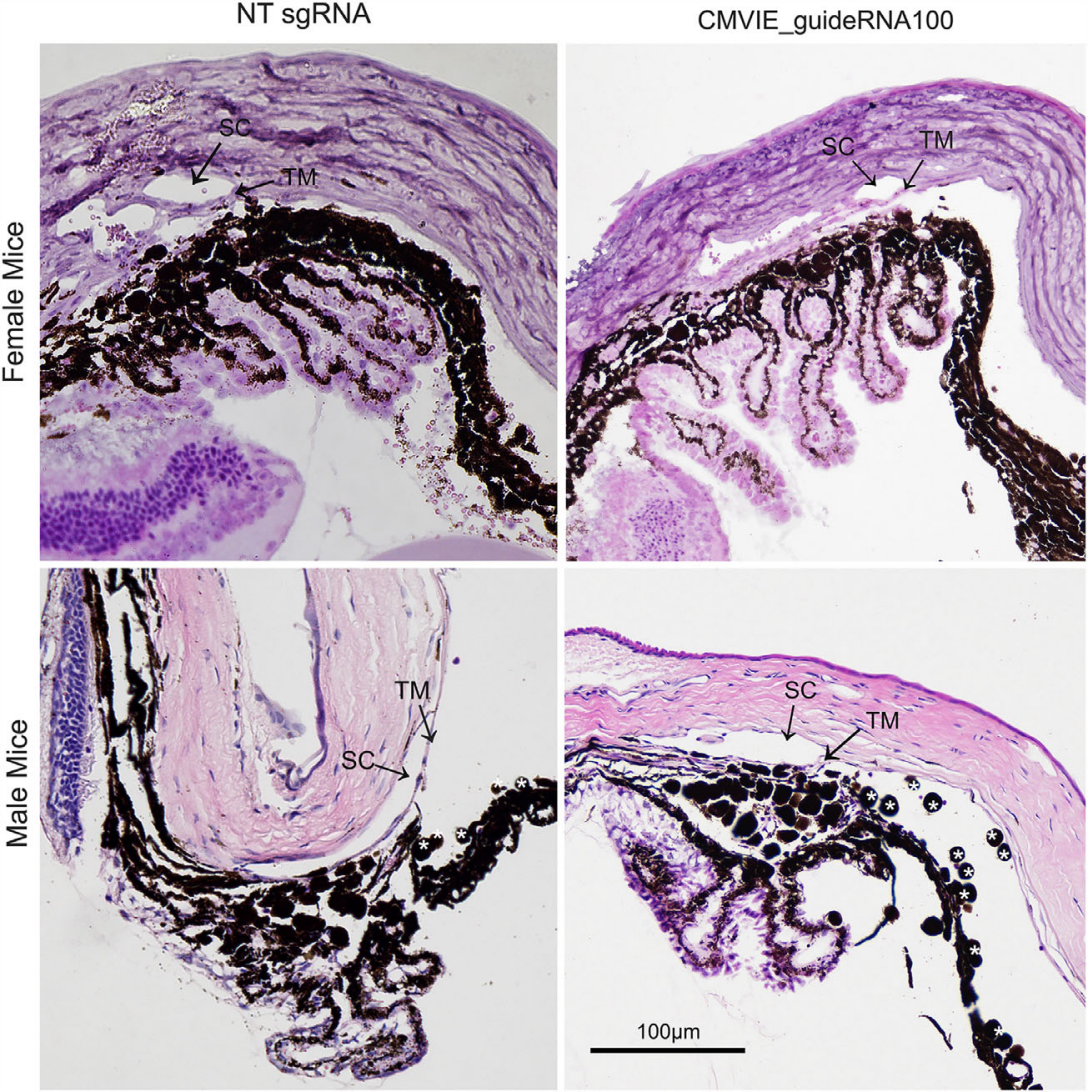
Morphology of the anterior chamber angle tissues of viral injected mice. Both male and female mouse eyes that received viral injection as described in Figure 5 were used for H&E staining. SC: Schlemm's canal. TM: trabecular meshwork. *: inflammatory cells.

H&E staining showed that the anterior chamber angle and the Schlemm's canal were open in NT sgRNA and CMVIE_guideRNA100 injected eyes. Also, there was no obvious inflammation in female eyes (Fig. 6, top row). In contrast, we observed some inflammation in some of the male eyes (Fig. 6, bottom row), which was likely due to higher adenoviral doses.

**Figure 7.**
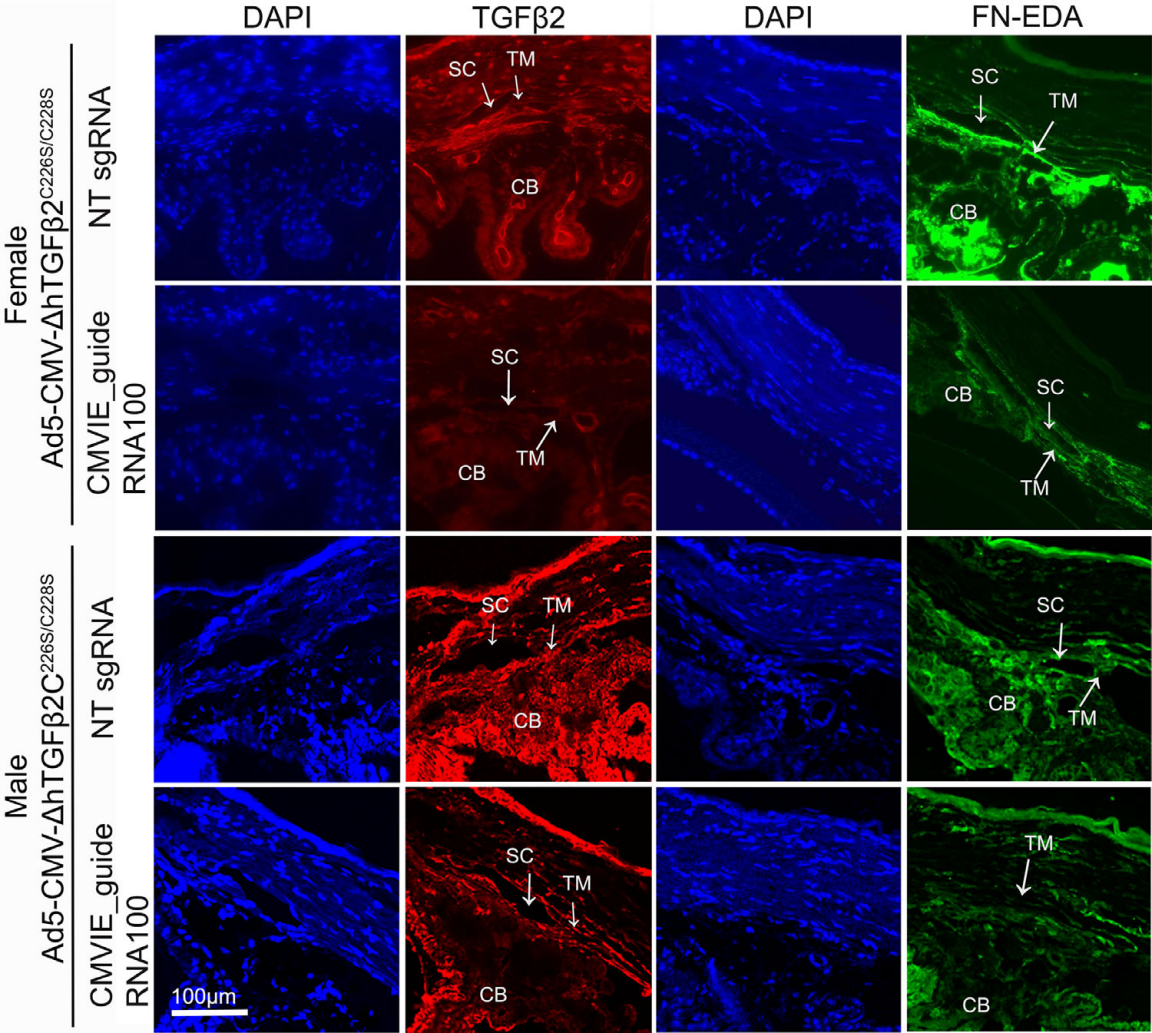
Inhibition of TGFβ2 and FN-EDA by CRISPR interference in mouse eyes. Both male and female mouse eyes that received viral injection as described in Figure 5 were used for immunostaining. TGFβ2: *red*; FN-EDA: *green*; DAPI: *blue*. TM: trabecular meshwork; SC: Schlemm's canal; CB: ciliary body.

Immunostaining showed a decrease in TGFβ2 and FN-EDA levels in the TM of the eyes injected with CMVIE_guideRNA100 compared to those injected with NT sgRNA (Fig. 7). FN-EDA was studied because FN has multiple isoforms while elevated FN-EDA expression plays a role in the glaucomatous TM.^25^

## Discussion

We found that the KRAB-dCAS9 and sgRNA CRISPR interference system was able to lower TGFβ2 levels in TM cells and tissues as well as inhibit OHT in TGFβ2 overexpressing mouse eyes. Studies showed that there is about twofold increase in TGFβ2 expression in the aqueous humor of POAG patients compared to that of the non-POAG cataract patients.^6^^,^^7^^,^^11^ Decreasing TGFβ2, instead of completely removing TGFβ2 (TGFβ2 plays a key role in ocular homeostasis) will help to treat POAG. Our study showed that CRISPR interference is a suitable technology for this purpose.

As described previously, histone acetylation may be a contributing factor to glaucomatous TGFβ2 elevation in the glaucomatous TM.^14^ There are several epigenetic approaches to lower TGFβ2 expression including histone acetyltransferase (HAT) inhibitors, RNA interference, and blocking antibodies. HAT inhibitors, like HDAC inhibitors, affect the entire genome and many enzymes in the cell due to the lack of specificity. RNA interference is a relatively mature technique. However, it attempts to *neutralize* all the already transcribed RNA rather than turn off RNA transcription. Therefore, RNA interference is presumably less ideal compared to CRISPR interference. The blocking antibodies have been studied in clinical trials for inhibiting fibrosis after antiglaucoma filtration surgeries to preserve the filtering bleb.^26^^,^^27^ However, the outcome was not ideal. Also, no clinical trials have been conducted for TGFβ2 blocking antibodies intraocularly.

The source of elevated intraocular TGFβ2 is not entirely clear. Although published studies showed that the glaucomatous TM tissue itself has more TGFβ2,^28^ all the intraocular tissues that have access to aqueous humor may contribute to TGFβ2 in addition to TM autocrine secretion. In this study, we successfully inhibited TGFβ2 expression in the TM. This inhibition may be due to inhibition of TGFβ2 produced by the TM as well as TGFβ2 produced by other intraocular tissues that flows with the aqueous humor and binds to the ECM and cells at the TM region since the Ad5 adenovirus may transduce some non-TM tissues.

Since aging is an important risk factor of POAG besides OHT, the relationship between TGFβ2 may provide insightful information. Tripathi and colleagues as well as Picht and colleagues both found that TGFβ2 levels did not change during aging.^6^^,^^29^ In contrast, Yamamoto and colleagues reported that there was a decrease of total TGFβ2 in the aqueous humor, but it was still higher in open angle glaucoma (OAG) eyes compared to non-OAG eyes.^30^ Although there is conflict in reports, it is clear that high TGFβ2 or relatively high TGFβ2 (assuming TGFβ2 decreases with age) is associated with glaucoma.

Also, since family history is another important risk factor of glaucoma, it would be interesting to find out if genetics plays a role in elevated TGFβ2 or TGFβ signaling. To our best knowledge, there has not been consensus in human genetic mutations of TGFβ2 or related genes which have clearly shown contributions to glaucomatous TM changes.

Besides inhibiting TGFβ2, an alternative strategy to suppress TGFβ signaling in the TM would be inhibiting TGFβ2 receptors. The advantage of this strategy is that the source of TGFβ2 is no longer a concern. The disadvantage is that the expression of the CRISPR interference system must be precisely located at the TM to be effective.

CRISPR interference is a very useful tool, and we believe that improvement in the following aspects would greatly facilitate its application:

a) sgRNA design. Unlike RNA interference, sgRNA design is strand dependent and strand direction needs to be considered. Also, there has been no consensus on where the sgRNA should target although some studies recommend targeting a small region spanning the transcriptional start site.^23^^,^^31^ With the increasing popularity of CRISPR interference, more web-based sgRNA design tools are now providing automated design for CRISPR interference sgRNAs.
b) Specificity. We have not determined the specificity of the CRISPR interference in detail in our study. The off-target effect of CRISPR has been well recognized and is very likely to affect the CRISPR interference system.
c) Complexity. Compared to siRNA and shRNA, CRISPR interference requires an additional component, the CAS9 enzyme. Since the size of the dCAS9 protein/gene (1368 amino acids or 4104 base pairs) plus the nuclear localization signal sequence and the modification enzyme (like KRAB) is relatively large, gene cloning is more difficult and vector selection is limited (such as adeno-associated viruses). Also, the efficiency of the system will be limited by dCAS9 expression and translocation.
d) Promoter selection. For TM cells and tissues, the CMV promoter has been widely used. However, we found that it did not perform well compared to the SFFV promoter although KRAB-dCAS9 was expressed. The mechanism(s) behind these findings needs further investigation.
e) Delivery. Since the CRISPR interference system is similar to the CRISPR system, theoretically, all available delivery approaches for CRISPR are suitable for CRISPR interference, including plasmids, viral vectors, physical delivery, and extracellular vesicles.^32^ In contrast, siRNA delivery is more convenient. In TM research, primary human TM cells can be transfected with siRNA, but no reports have shown that they can be transfected with an expression plasmid for gene overexpression, which is consistent with our experience. Therefore, to apply the CRISPR interference system in primary human TM cells, viral transduction seems to be the only choice.
f) Duration. Different from siRNA delivery, viral vector-mediated gene expression lasts from weeks to years depending on vector and tissue types. For the TM research, the most frequently used viral vectors are serum type 5 adenoviruses. Millar and colleagues reported that intravitreal injection of 1 × 10^7^ or 1 × 10^8^ PFU Ad5-GFP in male Balb/cJ mice resulted in GFP expression up to about 30 to 40 days, respectively.^33^ The lentivirus has limited reports. Balaggan and colleagues reported that intracameral injection of 3 × 10^6^ lenti-GFP vector resulted in GFP in the TM for at least 10 months.^34^ In this study, we observed an IOP lowering effect for at least 12 weeks.

Although our findings are encouraging, the main limitation of this study is that in our mouse model we inhibited exogenous TGFβ2 (mediated by the adenovirus). Since there has not been a suitable mouse model with spontaneously elevated TGFβ2, TGFβ2-induced OHT, and without development abnormalities (many TGFβ2 transgenic models have anterior segment dysgenesis), whether our system is also effective in suppressing endogenous TGFβ2 in vivo is unclear.

Regardless of potential technical difficulties, we believe that with the development of new technologies, the CRISPR inference system will have more and more applications in both research and gene therapy.

## Supplementary Material

Supplement 1

Supplement 2

Supplement 3
