# Supplementary material for: Using CRISPR Interference as a Therapeutic Approach to Treat TGFβ2-Induced Ocular Hypertension and Glaucoma
Source: Invest Ophthalmol Vis Sci. 2021 Sep 9;62(12):7. doi: 10.1167/iovs.62.12.7 (PMC8434756; doi:10.1167/iovs.62.12.7)
Supplement: Supplement 1 [file iovs-62-12-7_s001.pdf]

		Naive	NT sgRNA	TGFβ2 sgRNA 15	TGFβ2 sgRNA 23	TGFβ2 sgRNA 42	TGFβ2 sgRNA 43
Active TGFβ2	Sample 1	1.00	0.92	0.85	0.73	0.42	0.30
	Sample 2	1.00	0.96	1.27	1.30	0.85	0.61
	Sample 3	1.00	0.70	0.31	0.53	0.42	0.76
Total TGFβ2	Sample 1	1.00	1.12	0.80	1.54	0.76	0.92
	Sample 2	1.00	0.81	1.51	1.01	0.70	0.79
	Sample 3	1.00	1.94	1.80	1.13	0.64	0.61

Supplemental Table 1. Densitometry of the level of TGFβ2 in GTM3 cells.

GTM3 cells were transduced with the described vectors, and the proteins were used for WB. The active and total TGFβ2 protein bands in whole cell lysate blots were used for densitometry with normalization to GAPDH. Naïve controls were set at "1.00". N=3.
