# Supplementary material for: Using CRISPR Interference as a Therapeutic Approach to Treat TGFβ2-Induced Ocular Hypertension and Glaucoma
Source: Invest Ophthalmol Vis Sci. 2021 Sep 9;62(12):7. doi: 10.1167/iovs.62.12.7 (PMC8434756; doi:10.1167/iovs.62.12.7)
Supplement: Supplement 3 [file iovs-62-12-7_s003.pdf]

		NT sgRNA	CMVIE_guideRNA 47	CMVIE_guideRNA 100	CMVIE_guideRNA 195	CMVIE_guideRNA 468
Active TGFβ2	Sample 1	1.00	1.48	0.84	0.96	0.95
	Sample 2	1.00	0.63	0.52	0.74	0.95
	Sample 3	1.00	1.19	1.07	3.57	0.91
Total TGFβ2	Sample 1	1.00	1.05	0.36	1.02	1.08
	Sample 2	1.00	0.30	0.27	0.12	0.41
	Sample 3	1.00	0.39	0.20	0.77	0.35

Supplemental Table 3. Densitometry of the level of TGFβ2 in GTM3 cells transduced with Ad5-CMV-ΔhTGFβ2$^{C226S/C228S}$.

GTM3 were co-transduced with Ad5-CMV-ΔhTGFβ2$^{C226S/C228S}$ and the described vectors. The proteins were used for WB. The active and total TGFβ2 protein bands in whole cell lysate blots were used for densitometry with normalization to GAPDH. Nontargeting sgRNA (NT sgRNA) controls were set at “1.00”. N=3.
